# Supplementary material for: Sorting at embryonic boundaries requires high heterotypic interfacial tension
Source: Nat Commun. 2017 Jul 31;8:157. doi: 10.1038/s41467-017-00146-x (PMC5537356; doi:10.1038/s41467-017-00146-x)
Supplement: Supplementary file 2 — Supplementary Software 1 [file 41467_2017_146_MOESM2_ESM.zip › PottsModel/SrcPottsModel/doc/model/Constants.MetaManager.html]

Constants.MetaManager


---


|  |  |  |
| --- | --- | --- |
| **Overview** \| **Package** \| **Class** \| **Use** \| **Tree** \| **Deprecated** \| **Index** \| **Help** | |  |
| **PREV CLASS**   **NEXT CLASS** | **FRAMES**    **NO FRAMES**     **All Classes** |  |
| SUMMARY: NESTED \| FIELD \| CONSTR \| METHOD | DETAIL: FIELD \| CONSTR \| METHOD |  |


---


## model Class Constants.MetaManager

```
java.lang.Object
  model.Constants.Manager
      model.Constants.MetaManager
```

**Enclosing class:**: Constants

---

``` private class Constants.MetaManager extends Constants.Manager ```

Manages meta and statistical information related to this simulation.

---

| **Field Summary** | |
| --- | --- |
| `private  boolean` | `appendToCSV` |
| `private  boolean` | `areaStatisticTracked` |
| `private  boolean` | `automaticMode` |
| `private  java.lang.String` | `basename` |
| `private  boolean` | `cellEnergyStatisticTracked` |
| `private  boolean` | `csvSaved` |
| `private  int` | `csvWriteFrequency` |
| `private  boolean` | `debugMode` |
| `private  java.lang.String` | `description` |
| `private  boolean` | `dispersionIndexStatisticTracked` |
| `private  boolean` | `hblStatisticTracked` |
| `private  boolean` | `latticeSaved` |
| `private  int` | `latticeWriteFrequency` |
| `private  int` | `maxMcs` |
| `private  java.lang.String` | `name` |
| `private  boolean` | `perimeterStatisticTracked` |
| `private  boolean` | `snapshotsSaved` |
| `private  int` | `snapshotWriteFrequency` |
| `private  boolean` | `successfulSpinCopyAttemptsTracked` |
| `private  java.util.Date` | `timeStamp` |
| `private  java.lang.String` | `timeStampString` |

**Fields inherited from class model.Constants.Manager**

`prop`


**Constructor Summary**

`Constants.MetaManager(java.util.Properties p)`


| **Method Summary** | |
| --- | --- |
| `void` | `processProperties()` |
| `private  void` | `setName(java.lang.String newName)` |

**Methods inherited from class java.lang.Object**

`clone, equals, finalize, getClass, hashCode, notify, notifyAll, toString, wait, wait, wait`

**Field Detail**

### name

```
private java.lang.String name
```

---


### basename

```
private java.lang.String basename
```

---


### description

```
private java.lang.String description
```

---


### timeStamp

```
private java.util.Date timeStamp
```

---


### timeStampString

```
private java.lang.String timeStampString
```

---


### maxMcs

```
private int maxMcs
```

---


### debugMode

```
private boolean debugMode
```

---


### automaticMode

```
private boolean automaticMode
```

---


### snapshotsSaved

```
private boolean snapshotsSaved
```

---


### csvSaved

```
private boolean csvSaved
```

---


### latticeSaved

```
private boolean latticeSaved
```

---


### appendToCSV

```
private boolean appendToCSV
```

---


### snapshotWriteFrequency

```
private int snapshotWriteFrequency
```

---


### csvWriteFrequency

```
private int csvWriteFrequency
```

---


### latticeWriteFrequency

```
private int latticeWriteFrequency
```

---


### successfulSpinCopyAttemptsTracked

```
private boolean successfulSpinCopyAttemptsTracked
```

---


### hblStatisticTracked

```
private boolean hblStatisticTracked
```

---


### cellEnergyStatisticTracked

```
private boolean cellEnergyStatisticTracked
```

---


### dispersionIndexStatisticTracked

```
private boolean dispersionIndexStatisticTracked
```

---


### areaStatisticTracked

```
private boolean areaStatisticTracked
```

---


### perimeterStatisticTracked

```
private boolean perimeterStatisticTracked
```


**Constructor Detail**

### Constants.MetaManager

```
public Constants.MetaManager(java.util.Properties p)
```


**Method Detail**

### processProperties

```
public void processProperties()
```

:   **Specified by:**: `processProperties` in class `Constants.Manager`

---


### setName

```
private void setName(java.lang.String newName)
```


---


|  |  |  |
| --- | --- | --- |
| **Overview** \| **Package** \| **Class** \| **Use** \| **Tree** \| **Deprecated** \| **Index** \| **Help** | |  |
| **PREV CLASS**   **NEXT CLASS** | **FRAMES**    **NO FRAMES**     **All Classes** |  |
| SUMMARY: NESTED \| FIELD \| CONSTR \| METHOD | DETAIL: FIELD \| CONSTR \| METHOD |  |


---
